# On the performance of tests for the detection of signatures of selection: a case study with the Spanish autochthonous beef cattle populations

**DOI:** 10.1186/s12711-016-0258-1

**Published:** 2016-10-28

**Authors:** Aldemar González-Rodríguez, Sebastián Munilla, Elena F. Mouresan, Jhon J. Cañas-Álvarez, Clara Díaz, Jesús Piedrafita, Juan Altarriba, Jesús Á. Baro, Antonio Molina, Luis Varona

**Affiliations:** 1 Departamento de Anatomía, Embriología y Genética, Universidad de Zaragoza, 50013 Saragossa, Spain; 2 Departamento de Producción Animal, Facultad de Agronomía, Universidad de Buenos Aires, 1417 Buenos Aires, Argentina; 3 Grup de Recerca en Remugants, Departament de Ciència Animal i dels Aliments, Universitat Autònoma de Barcelona, 08193 Bellaterra, Barcelona, Spain; 4 Departamento de Mejora Genética Animal, INIA, 28040 Madrid, Spain; 5 Instituto Agroalimentario de Aragón (IA2), 50013 Saragossa, Spain; 6 Departamento de Ciencias Agroforestales, Universidad de Valladolid, 34004 Palencia, Spain; 7 MERAGEM, Universidad de Córdoba, 14071 Córdoba, Spain

## Abstract

**Background:**

Procedures for the detection of signatures of selection can be classified according to the source of information they use to reject the null hypothesis of absence of selection. Three main groups of tests can be identified that are based on: (1) the analysis of the site frequency spectrum, (2) the study of the extension of the linkage disequilibrium across the length of the haplotypes that surround the polymorphism, and (3) the differentiation among populations. The aim of this study was to compare the performance of a subset of these procedures by using a dataset on seven Spanish autochthonous beef cattle populations.

**Results:**

Analysis of the correlations between the logarithms of the statistics that were obtained by 11 tests for detecting signatures of selection at each single nucleotide polymorphism confirmed that they can be clustered into the three main groups mentioned above. A factor analysis summarized the results of the 11 tests into three canonical axes that were each associated with one of the three groups. Moreover, the signatures of selection identified with the first and second groups of tests were shared across populations, whereas those with the third group were more breed-specific. Nevertheless, an enrichment analysis identified the metabolic pathways that were associated with each group; they coincided with canonical axes and were related to immune response, muscle development, protein biosynthesis, skin and pigmentation, glucose metabolism, fat metabolism, embryogenesis and morphology, heart and uterine metabolism, regulation of the hypothalamic–pituitary–thyroid axis, hormonal, cellular cycle, cell signaling and extracellular receptors.

**Conclusions:**

We show that the results of the procedures used to identify signals of selection differed substantially between the three groups of tests. However, they can be classified using a factor analysis. Moreover, each canonical factor that coincided with a group of tests identified different signals of selection, which could be attributed to processes of selection that occurred at different evolutionary times. Nevertheless, the metabolic pathways that were associated with each group of tests were similar, which suggests that the selection events that occurred during the evolutionary history of the populations probably affected the same group of traits.

**Electronic supplementary material:**

The online version of this article (doi:10.1186/s12711-016-0258-1) contains supplementary material, which is available to authorized users.

## Background

The evolutionary history of animal populations involves both natural and artificial selection. These processes not only affect the allelic frequencies at causal polymorphisms, but also the surrounding genomic regions due to the so-called “hitchhiking” effect. Thus, they may leave detectable signals on the structure of the genome that can be identified by using appropriate procedures [1, 2].

The vast majority of the procedures used to detect signatures of selection [2] is based on the null hypothesis of absence of selection, which relies on the neutral model of evolution [3]. In fact, these procedures can be classified according to the source of information they use to reject the null hypothesis. Based on the literature [2], three main groups of tests can be identified: the first group is based on the analysis of the site frequency spectrum [4–6], the second group focuses on the study of the extension of the linkage disequilibrium across the length of the haplotypes that surround a polymorphism [7, 8], and the third group is based on several measures of differentiation among populations [9–11]. In addition, the results of all these tests can be affected to some degree by demographic events and by the ascertainment bias caused by the procedure used to select the single nucleotide polymorphisms (SNPs) for the genotyping chip [12]. Thus, the results of each test may not be fully consistent with each other [13], which has led to propose strategies for summarizing results into a single statistic that either does [13] or does not [14, 15] account for the correlations between the results from different methods.

The aim of our study was to compare the performance of a subset of these procedures by using a dataset on seven autochthonous beef cattle populations (Asturiana de los Valles, Avileña-Negra Ibérica, Bruna dels Pirineus, Morucha, Pirenaica, Retinta and Rubia Gallega) which share close genetic relationships between them [16]. A second objective was to identify candidate genes and/or metabolic processes that are associated with the regions involved in the selection processes that occurred during the evolution of these populations.

## Methods

### Animals and sample size

A total of 171 sire/dam/offspring triplets were collected from seven Spanish beef cattle populations, including Asturiana de los Valles (AV, n = 25), Avileña-Negra Ibérica (ANI, n = 24), Bruna dels Pirineus (BP, n = 25), Morucha (Mo, n = 24), Pirenaica (Pi, n = 24), Retinta (Re, n = 24) and Rubia Gallega (RG, n = 24) breeds. The selected parents were chosen as unrelated as possible to fully represent the diversity of the populations.

### SNP genotyping and phasing

Genomic DNA was extracted by standard protocols. High-density SNP genotyping was performed at a commercial laboratory (Xenética Fontao, Lugo, Spain) by using the BovineHD BeadChip (Illumina Inc, USA) according to the manufacturer’s protocol; this HD chip is designed to genotype 777,962 SNPs. The SNPs that were retained for our study were located on autosomal chromosomes at a single position. Additional requirements were a Mendelian error rate lower than 0.05, and SNP and individual call rates higher than 0.95. Quality control was performed by using PLINK software [17] and finally, 703,707 SNPs that covered 2,510,606 kb were available for the analyses with on average one SNP per 3.567 kb. Haplotypes for the parental chromosomes were derived with Beagle software [18] using the “TRIO” option.

### Detection of signatures of selection

The data were analysed using the following procedures for the detection of signatures of selection.

#### Tajima

The procedure that was developed by Tajima [4] compares two statistics to estimate the scaled mutation rate. The first statistic $\left( {\theta_{\pi } } \right)$ is based on the number of segregating sites within a genomic region and the second $\left( {\theta_{\kappa } } \right)$ is the average heterozygosity at segregating sites in the sample. The standardized difference between these two values, $D = \theta_{\pi } - \theta_{\kappa } ,$ is used to infer departures from neutrality. Theoretically, if $D < 0$ either the population has suffered expansion after a recent bottleneck or a recent selective sweep has taken place; on the contrary if $D > 0,$ the population has either experienced a sudden population contraction or is under balancing selection. The analysis was performed over sliding windows of 100 SNPs by using own software.

#### Fay and Wu

This procedure [6] calculates the following statistic $D = \theta_{\pi } - \theta_{H} ,$ where $\theta_{H}$ depends on the number of sites at which a derived allele is present within a genomic region. In the analysis, the ancestral alleles were extracted from the study of Rocha et al. [19]. This test was computed over sliding windows of 100 SNPs by using own software.

#### Fu and Li

This procedure [5] is based on counting the number of singletons or alleles present in only one phase. The rationale is that a selection process will extend time to coalescence so that a larger number of mutations may take place in new or external branches of the tree and thus appear only once in the observed sample. As before, the analysis was performed over sliding windows of 100 SNPs by using own software.

#### iHS

This procedure [8] calculates the ratio of the integrated haplotype score (*iHH*) for the ancestral allele and the derived allele at a given SNP. The *iHH* is the integral (area) of the observed decay of the *EHH* (extended haplotype homozygosity) as defined by Sabeti et al. [7]. As in the previous test, ancestral alleles were extracted from Rocha et al. [19]. The *iHS* was calculated with the *selscan* software [20] using the parameters recommended by the authors. For further calculations, we used the |*iHS|.*

#### nSL

This procedure was recently presented by Ferrer-Admetlla et al. [21]. The procedure of calculation is similar to *iHS*, but replaces *IHH* by an alternative statistic (*SL*) that measures the length of a segment of haplotype homozygosity in terms of segregating sites. The main advantage of *nSL* over *iHS* is that it uses segregating sites as a measure of distance, while *iHS* needs the recombination distance. Thus, the *iHS* is more sensitive to recombination rate [21]. The analysis used the same parameters as in the *iHS* test and own software. As before, we used |nSL|.

#### *H*12

This method was recently proposed [22] with the *H*12 statistic being defined as:

$$H12 = \left( {p_{1} + p_{2} } \right)^{2} + \mathop \sum \limits_{j > 2} p_{j}^{2} ,$$

where $p_{j}$ is the frequency of the *j*th most common haplotype in the population. Here, the frequencies of the first and second most common haplotypes were combined into a single frequency. The calculation was performed over sliding windows of 100 SNPs by using own software.

#### Fixation index (*F*_ST_)

This procedure was described by Wright [9] and is the most classical approach to study the pattern of differentiation between populations. The fixation index $F_{\text{ST}}$ is calculated for each SNP and for each pair of populations as $F_{\text{ST}} = \left( {H_{O} - H_{E} } \right) /H_{E} ,$ where $H_{O}$ and $H_{E}$ are the observed and expected heterozygosities, respectively. Estimates for $F_{\text{ST}}$ were averaged over sliding windows of 100 SNPs and assigned to the central SNP in each window. The procedure was computed with own software. Finally, the results for each population were computed by averaging the paired $F_{\text{ST}}$ estimates with the other six populations.

#### Selestim

This procedure [10] assumes a hierarchical Bayesian model to distinguish selected polymorphisms from the background of neutral (or almost neutral) polymorphisms and also to estimate the intensity of selection in each population. The model assumes a binomial distribution of the allele counts at each locus and for each population, and the prior distribution of allelic frequencies is modeled under the assumption of a stationary density of the diffusion process [10]. The model is implemented by using a Markov chain Monte Carlo method. SelEstim software (http://www1.montpellier.inra.fr/CBGP/software/selestim/) was used for this purpose with the standard parameters that are recommended by the authors. Among the outputs provided by the Selestim approach, we extracted the $\sigma_{ij}$ parameter [10], which represents the coefficient of selection for the *i*th subpopulation and the *j*th locus.

#### XP-CLR

This approach [23] assumes that the allele frequencies of two populations that diverge from an ancestral population follow a Gaussian distribution for which the variance contains information on the history of the populations since they split. Under the assumption that the evolutionary process is reversible, the procedure defines the distribution of allelic frequencies in the first population (reference) given the allele frequencies in the second population (objective). We calculated *XP*-*CLR* by taking each pair of populations as objective and reference with the software *XPCLR* (http://genetics.med.harvard.edu/reich/Reich_Lab/Software.html). Then, we averaged the six available tests for each population that was treated as an objective population, to infer the signatures of selection for each breed.

#### XP-EHH

This approach [7] is also computed for each pair of populations. For each population, as in the *iHS* test, it calculates the *EHH* between a core SNP and a set of SNPs within a predefined genomic interval and integrates it with respect to genetic distance to calculate the integrated haplotype score (*IHH*) for populations A and B. Then, the statistic is computed as $XPEHH_{AB} = \ln \left( {IHH_{A} /IHH_{B} } \right).$ As previously, we computed this statistic for each SNP and each pair of populations and the results were averaged over the six comparisons to generate a unique result for each population. We used the software *selscan* [20] with the parameters that are recommended by the authors.

#### VarLD

This procedure [24] evaluates the magnitude of the differences in linkage disequilibrium between a pair of populations. It calculates the linkage disequilibrium as the correlation coefficient between pairs of SNPs within a genomic region and creates a matrix of those correlations for each population. Then, it evaluates the differences between the matrices of both populations as the difference between its eigenvalues. The procedure was computed using the software VarLD [25] over sliding windows of 100 SNPs.

For all the above-described methods, we used the empirical distribution of the results generated along the genome as the null distribution of the test, in order to reduce the possible effects of the demographic history or the ascertainment bias. The underlying hypothesis is that, on average, both demographic events and ascertainment bias affect all the genome in a similar way, and thus, deviations or extreme values of the empirical distribution could be understood as signals of selection events.

### Summary of signals of selection

In order to detect communalities and summarize the results of the 11 procedures for ease of interpretation, we normalized these results for each SNP using a logarithm transformation to make the scale of the different results comparable and, then, we calculated the correlation between the logarithms (or the negative of logarithms for the Tajima, Fu and Li and Fay and Wu procedures) for the 703,707 SNPs. In a confirmatory analysis, provided that the methods used to detect signatures of selection were classified into three groups, we performed a factor analysis restricted to a subspace of three axes using a *varimax* rotation [26]. The analysis was done with *R* [27] by using the function *principal*() included in the package *psych*.

### Selection of candidate genes

First, we identified candidate genes based on the empirical distribution of the output of the three canonical axes of the factor analysis. Thus, we defined a very strict threshold by selecting the genomic 1-Mb regions with at least 25 SNPs that were in the top 0.1% of the results for each axis. Then, we used the *Ensembl*-*Biomart* database to identify the genes that were present in those genomic regions and compared our results with those in the literature to identify potential candidate genes for selection in the bovine populations.

### Enrichment analysis

Finally, in order to obtain a clearer picture of the metabolic pathways that were affected by the selection processes, we identified the genomic regions that were above the top 5% of each canonical axis. The objective of the relaxation of the empirical threshold was to capture softer signals of selection. With these selected genomic regions for each canonical axis, we used the software WebGestalt [28] (http://bioinfo.vanderbilt.edu/webgestalt/) by setting the *Homo sapiens* genome as the reference genome. In addition, we used a hypergeometric p value to correct for multiple-testing. The results included the top 10 pathways (WikiPathways).

## Results and discussion

### Summarizing footprints of selection detected by 11 procedures

**Fig. 1:**
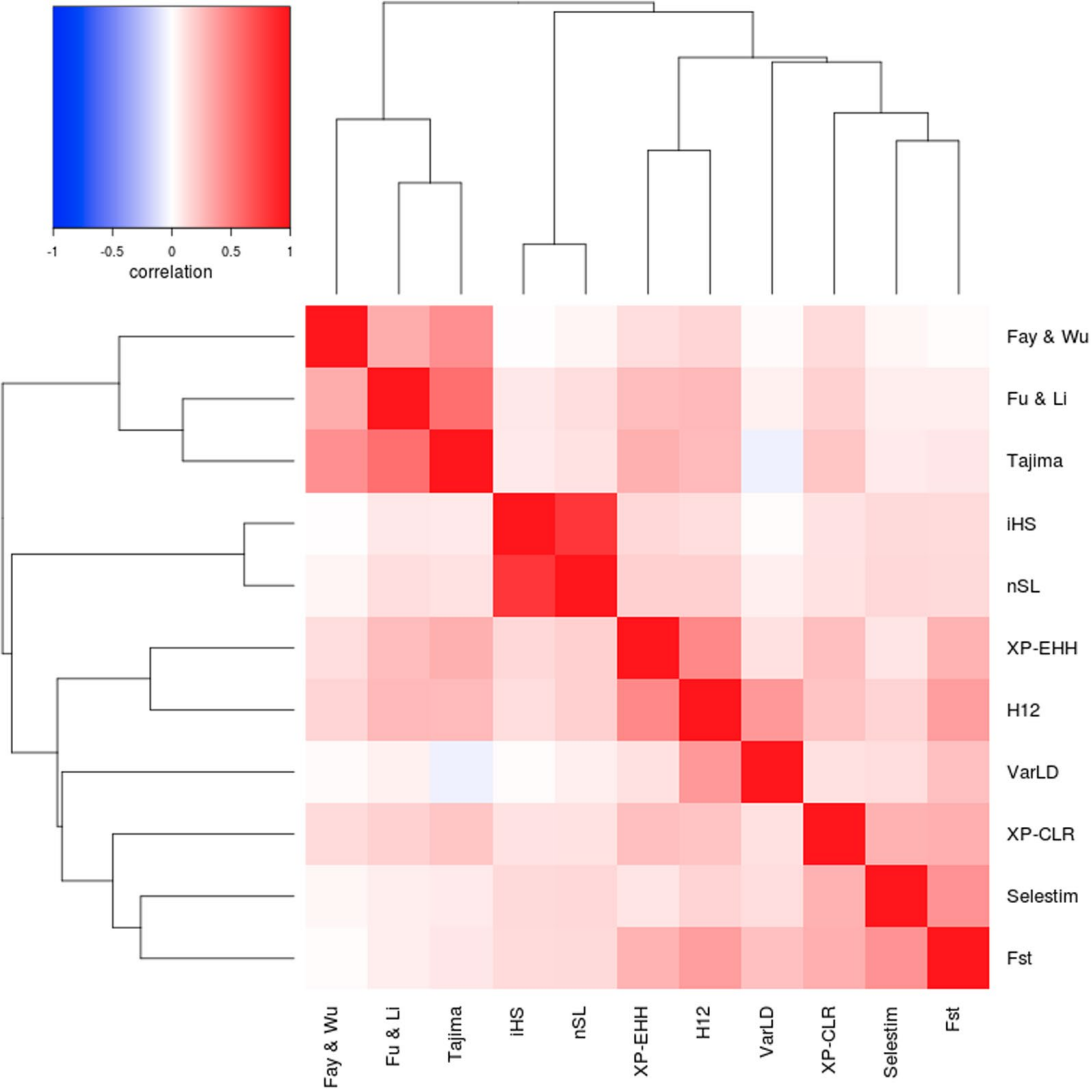
Heatmap of the correlations between logarithms of the results of the 11 tests applied for the detection of signatures of selection and their clustering

A large set of procedures is available for the identification of footprints of selection across the genome [2]. Most of these procedures are based on the rejection of the null hypothesis of absence of selection based on the neutral theory of evolution [3]. However, each of these methods calculates a different statistic to test this hypothesis. In addition, they are influenced to varying degrees by demographic history and ascertainment bias caused by the selection of SNPs [12]. Thus, it is expected that each test provides a different output as confirmed by the correlations between the results obtained by the 11 procedures used in this study (Fig. 1) and by the Manhattan plots generated with the results for each test and population (see Additional file 1: Figure S1, Additional file 2: Figure S2, Additional file 3: Figure S3, Additional file 4: Figure S4, Additional file 5: Figure S5, Additional file 6: Figure S6, Additional file 7: Figure S7, Additional file 8: Figure S8, Additional file 9: Figure S9, Additional file 10: Figure S10, Additional file 11: Figure S11).

In order to summarize the signals of selection that were detected by the 11 tests, there are procedures to condense such results into a single statistic by using Bayes factors [14] or a combination of p values [13, 15]. However, these strategies imply that the signals of selection that are captured by the different methods are comparable. Nevertheless, as the definition of the null hypothesis varies between tests, the signals of selection identified by each procedure may correspond to different types of selection events. In fact, some authors [29] pointed out that within-population haplotype length methods [8, 21] can detect only very recent selection processes, because they become ineffective when the selected alleles reach fixation or are very close to fixation. The same authors [29] indicated that signals of selection that are based on a reduction of genetic diversity [4, 5] persist for a longer period of time and these methods can detect older signals of selection, while tests that are based on population differentiation [9, 10] occupy an intermediate position.

In this study, the correlations of the absolute logarithm of the results between the 11 methods used were low or even negative (Fig. 1). However, there are some remarkable exceptions such as the correlations between Tajima and Fu and Li, and iHS and nSL tests, that were remarkably high. It should be noted that the Tajima and Fu and Li tests are both based on the analysis of the site frequency spectrum, and that the nSL test is just a modification of iHS where the map distance is replaced by the number of segregating sites [21]. A more detailed analysis of the structure of these correlations allows to identify three main groups of tests, one group based on the site frequency spectrum (Tajima, Fu and Li, Fay and Wu); a second group based on the haplotype length (iHS and nSL), and a third group that focuses on the differentiation between populations (*F*_ST_, SelEstim and XP-CLR). The remaining tests (VarLD, H12 and XP-EHH) are in an intermediate position between the latter two, although slightly closer to tests based on population differentiation. Such a structure of the correlations between the results of these methods indicates that the implementation of a factor analysis, as suggested by Simianer et al. [30], could be appropriate to summarize the results into a few canonical axes. In addition, as described below, each axis was associated with signatures of selection of a different kind. In particular, we applied a factor analysis restricted to three canonical axes using a varimax approximation [26] that explains up to the 56% of variation.

**Fig. 2:**
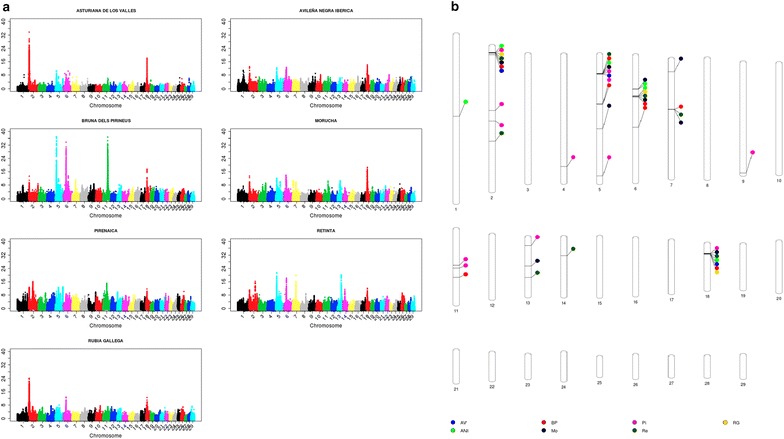
Manhattan plots for the results of the first axis (**a**) and genomic regions identified with at least 25 SNPs within the top 0.1% of the results (**b**)

**Fig. 3:**
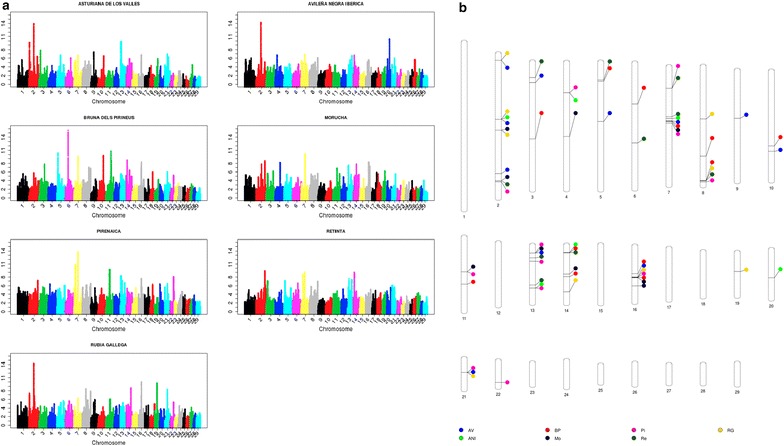
Manhattan plots for the results of the second axis (**a**) and genomic regions identified with at least 25 SNPs within the top 0.1% of the results (**b**)

**Fig. 4:**
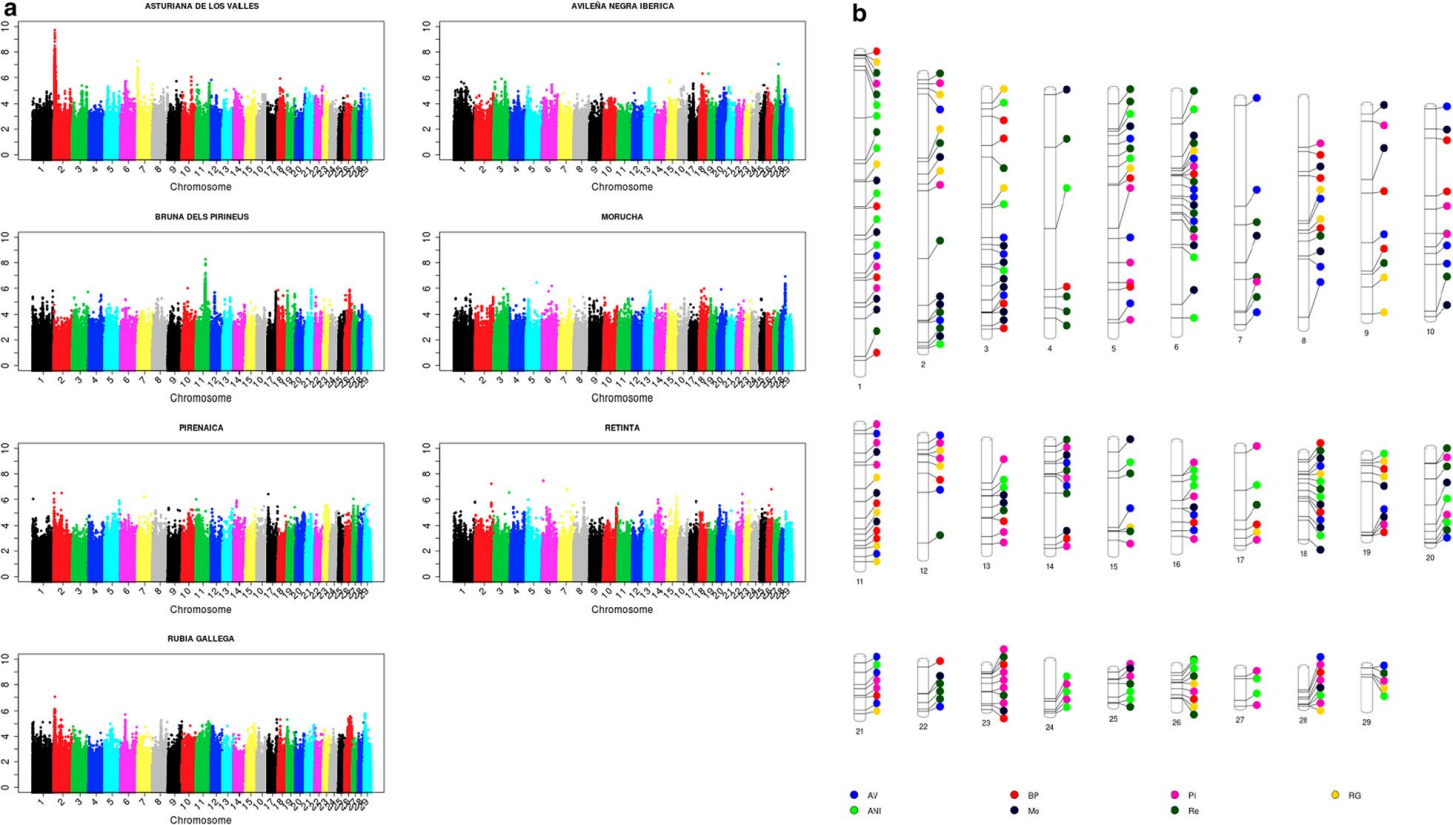
Manhattan plots for the results of the third axis (**a**) and genomic regions identified with at least 25 SNPs within the top 0.1% of the results (**b**)

**Fig. 5:**
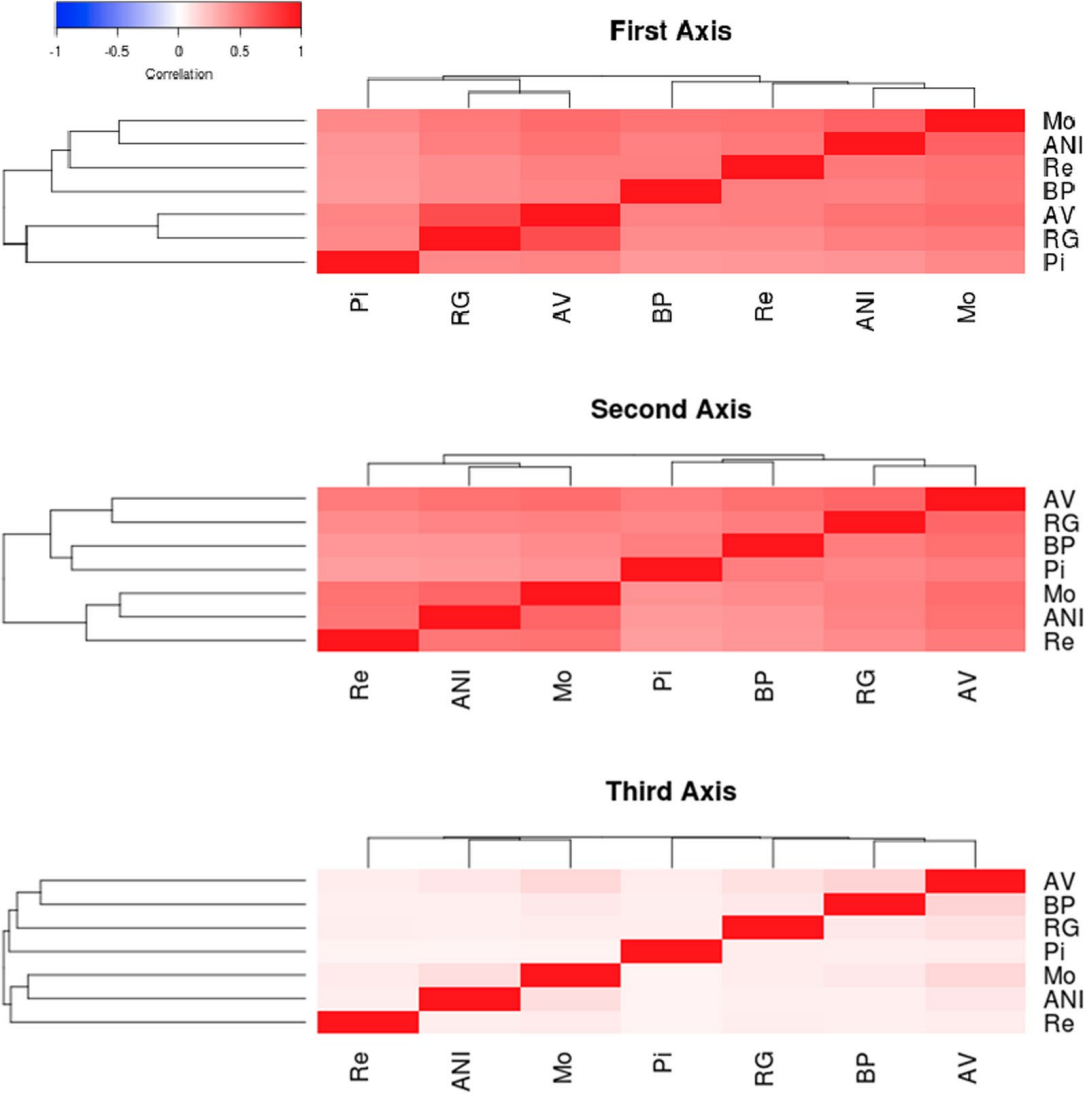
Correlations of the results from the first, second and third canonical axes between populations

Table 1 shows the loadings for the canonical axes that resulted from the factor analysis for each of the 11 tests used to detect selection signatures. Moreover, Table 1 presents the correlations between the canonical axes and each specific test, which are fully consistent with the results of the correlations presented in Fig. 1. The first axis explains 20% of the variation and shows a high correlation with the procedures based on the analysis of population differentiation (*F*_ST_, SelEstim, XP-EHH, XP-CLR and VarLD) and *H*12; the second axis explains 19% of the variation and is correlated with the methods based on the site frequency spectrum (Tajima, Fu and Li, and Fay and Wu); and, finally, the third axis is strongly correlated with methods based on the extension of linkage disequilibrium or haplotype length (iHS and nSL) and explains 17% of the total variation. For each test, the three axes explain between 32 (XP-CLR) and 90% (iHS) of the variation. The Manhattan plots of the results that relate to the three canonical axes are in Figs. 2, 3 and 4. The first two axes presented a higher level of shared signals between populations (see Figs. 2, 3) whereas the results of the third axis were, in general, breed-specific. This statement is supported by the results in Fig. 5, which shows the correlations of the results obtained for the first, second and third canonical axes between populations. An average correlation of 0.50 was found for the first axis [ranging from 0.39 (BP and Re) to 0.71 (AV and RG)]. Furthermore, the second axis also showed high correlations between populations that ranged from 0.37 (Re and Pi) to 0.60 (AV and RG) with an average of 0.49. On the contrary, the correlations for the third axis were lower with an average value of 0.08 and ranged from 0.05 (Pi and Re) to 0.16 (AV and BP). In addition, the structure of the correlations (Fig. 5) confirmed the classification of the populations into two main clusters, one composed by the ANI, Mo and Re populations and the other by the Pi, BP, RG and ANI populations, as previously reported by Cañas-Álvarez et al. [16] based on distance measures and admixture analysis.

**Table 1:** Weights in the factor analysis with, between parentheses, the correlation between the results of each test and the canonical axis, and percentage of variance explained by the three axes

Method	First axis	Second axis	Third axis	% variance
Tajima	−0.07 (0.07)	0.42 (0.85)	−0.01 (0.06)	73
Fu-Li	−0.08 (−0.02)	0.35 (0.68)	−0.05 (−0.04)	47
Fay-Wu	−0.05 (0.09)	0.38 (0.77)	−0.00 (0.07)	61
Selestim	0.28 (0.58)	−0.10 (−0.05)	0.02 (0.13)	36
XPCLR	0.22 (0.51)	0.06 (0.24)	−0.02 (0.06)	32
H12	0.29 (0.67)	0.08 (0.32)	−0.04 (0.06)	55
IHS	−0.06 (0.07)	−0.04 (0.03)	0.53 (0.95)	90
NSL	−0.04 (0.11)	−0.02 (0.07)	0.52 (0.94)	89
*F*_ST_	0.38 (0.77)	−0.10 (−0.02)	−0.03 (0.08)	60
XP-EHH	0.17 (0.47)	0.14 (0.40)	0.01 (0.13)	40
VarLD	0.31 (0.59)	−0.11 (−0.09)	−0.10 (−0.08)	36

As in the study of Sabeti et al. [29], our results may indicate that old selection or adaptation processes that occurred before breed differentiation or during speciation were only detected by the site frequency spectrum methods that are associated with the second canonical axis. The signals of selection that were generated by later isolation and recent selection events within the populations are identified by the differentiation methods, which are linked to the first canonical axis. Finally, the haplotype length methods, summarized in the third canonical axis, identified more recent and, in general, less intense selection events that are mostly specific to each population. The absence of regions with strong recent signals of selection agrees with the postulate that artificial selection processes do not leave relevant signatures of selection [31]. The main reason of this absence can be due to the polygenic nature of most of the traits associated with current selection processes [32–34] or to the effect of epistasis [35].

In fact, the most remarkable signal of selection from this third axis was identified on chromosome 2 around the *myostatin* (*MTSN*) gene (between 6,213,566 and 6,220,196 bp) in the AV population, where double-muscling is included as a criterion of selection in its breeding program. This specific genomic region can be used to illustrate the timing of the signatures of selection that were detected by each group of methods. In the AV population, two large signatures of selection were detected with the first and third canonical axes, respectively. The first axis is related to processes that were involved in the creation of the breed and the third axis to recent selection. In addition, a large signature of selection associated with the first canonical axis in the RG population was observed. However, in this population, there is no relevant signature in the results of the third axis. This result is consistent with several previous studies [36] that reported the presence of haplotypes associated with double-muscling also in the RG population. This may indicate that some degree of selection around this gene may have occurred during the process of breed formation, but the current breeding program does no longer put any selection pressure on double-muscling. Finally, the results from the second canonical axis are less relevant (AV) or even absent (RG), which indicates that, for this group of tests, the selection effects may be diluted because a larger number of generations without selection is considered.

### Candidate genes and metabolic paths

The results of the first axis (Fig. 2) allowed us to highlight seven relevant genomic regions on *Bos taurus* chromosome (BTA) 2 (between 1,047,347 and 11,899,039 bp), BTA5 (between 15,920,995 and 20,321,882 bp), BTA6 (between 37,853,912 and 41,160,000 bp), BTA7 (between 47,276,124 and 47,745,164 bp), BTA11 (between 65,077,840 and 72,203,248 bp), BTA13 (between 57,430,392 and 57,754,760 bp) and BTA18 (between 12,675,262 and 16,202,289 bp). In some cases, these genomic regions were extremely large, because of strong signatures of selection such as those on BTA2 for the AV and RG populations or on BTA5, BTA6 and BTA11 for the BP population. Such huge signatures of selection imply that large genomic regions included SNPs that were associated with results above the top 0.1% of the empirical distribution along the genome. However, the localization of the strongest signals within each genomic region and for each population allowed us to narrow down the genomic regions (Fig. 2b), which are similar to those reported in a previous study on the differentiation between populations [37]. These regions included well-known genes that were previously reported as potential candidates of selection signatures in cattle [38], such as *MTSN* (*myostatin*) on BTA2, suggested in several beef cattle populations [39–41], *KIT*-*LG* (*kit*-*ligand*) on BTA5 with a very large peak in the BP population, *MC1R* (*melanocortin 1 receptor*) on BTA18, which controls the production of eumelanin (black) or pheomelanin (red) pigments [42] and appears to be relevant in populations with black (AV and Mo) or red (Re) coat color. Moreover, it should be also highlighted that the region on BTA6 that includes *LAP3* (*leucine aminopeptidase3*), *LCORL* (*ligand dependent nuclear receptor corepressor*-*like*) and *NCAPG* (*non*-*SMC condensing I complex, subunit G*) and was identified in two meta-analyses [38, 43] as one the genomic regions that is most frequently identified with signatures of selection in the bovine genome. The genomic region identified on BTA7 includes the *CAMLG* (*calcium modulating ligand*) and *TCF* (*transcription factor 7*) genes, which are close to a strong signature of selection that was reported by Gautier [44] and is associated with the *VDAC1* (*voltage*-*dependent anion*-*selective channel protein 1*) gene. A strong signature of selection was observed for the genomic region on BTA13, in the Re population, where is located the *END3* (*endothelin 3*) gene that plays a role in melanocyte development [45] and was recently associated with piebald pattern [44]. Finally, there is a very strong signature of selection on BTA11 for the BP population, where the closest gene to the maximum signal is *BMP10* (*bone morphogenetic protein 10*). Within this genomic region on BTA11, some authors [46, 47] identified signatures of selection and several genes that could be associated with fertility: *PROKR1* (*prokineticin receptor 1*), *GFPT1* (*glutamine*-*fructose*-*6*-*phosphate transaminase 1*), *GMCL1* (*germ cell*-*less spermatogenesis associated 1*), *PCBP1* (*poly*(*rC*) *binding protein 1*) and *EHD3* (*EH*-*domain containing 3*).

The results of the second canonical axis (Fig. 3) confirmed some of the signals of selection that were detected in the first axis, but also revealed several new ones that are shared by several populations and located on five chromosomes: BTA2 (between 61,684,232 and 62,199,344 and between 72,158,144 and 73,356,296 bp), BTA7 (between 20,612,988 and 21,163,812 bp), BTA13 (between 11,860,881 and 12,062,522 bp), BTA16 (between 44,612,592 and 45,846,144 bp) and BTA21 (between 32,207,264 and 32,414,316 bp). Previously, two meta-analyses [38, 43] showed that these regions were also associated with signatures of selection in other populations. Among the genes included in these regions, some of them may be good candidates for being affected by selection i.e.: (1) genes that are related to energy balance and homeostasis: *R3HGM1* (*R3H domain containing 1*) on BTA2 [48]; *CAMK1D* (*calcium/calmodulin*-*dependent protein kinase ID*) on BTA13 [49]; and *SLC25A33* (*solute carrier family 25* (*pyrimidine nucleotide carrier*)*, member 33*) and *SLC2A5* (*solute carrier family 2* (*facilitated glucose/fructose transporter*), *member 5*) on BTA16 [49, 50]; (2) *PLIN5* (*perilipin 5*) on BTA7, which is involved in the regulation of lipid metabolism in rat and pigs [51, 52]; (3) *SCAPER* (*s*-*phase cyclin A*-*associated protein in the endoplasmic reticulum*) on BTA21, which regulates cell cycle progression [53]; and (4) there are two other signatures of selection that are worth noting i.e. one detected for the RG population on BTA19 (between 27,941,270 and 28,571,032 bp) where *ALOX15B* (*arachidonate 15*-*lipoxygenase, type B*) and *ALOX12B* (*arachidonate 12*-*lipoxygenase, 12R type*) are located and are involved in immune response [54], and one for the ANI population on BTA20 (between 40,854,136 and 40,996,384 bp) where the *NPR3* (*natriuretic peptide receptor 3*) gene is located, which is related with cattle stature [55].

Although less relevant, the results of the third canonical axis (Fig. 4) confirm the signatures of selection around the *MTSN* gene for the AV, RG and Pi populations, and around the complex *LAP*–*LCORL*–*NCAG* for the BP population. There are several other interesting signatures of selection such as those located on BTA27 (between 36,466,580 and 40,862,444 bp) and BTA28 (between 41,643,416 and 45,215,488 bp) for the ANI and Mo populations, respectively. The first genomic region includes the *IKBKB* (*inhibitor of kappa light polypeptide gene enhancer in B*-*cells, kinase beta*), *DKK4* (*dickkopf WNT signaling pathway inhibitor 4*) and *VDAC3* (*voltage*-*dependent anion*-*selective channel protein 3*) genes that are associated with immune response to *trypanosoma* infection in African populations [56], and the second region contains the *ALOX5* (*arachidonate 5*-*lipoxygenase*) and *RASSF4* (*ras association* (*RalGDS/AF*-*6*) *domain family member 4*) genes, that are related with growth [57] and feed conversion [58], respectively.

Finally, we performed a pathway enrichment analysis [59] for the identified genomic regions by applying a less restrictive criterion (top 5%). The objective of this analysis was to identify a larger number of genomic regions associated with signatures of selection although with less strong signals. The results of the top 10 pathways that were identified by the enrichment analysis are in Table 2. In general, the pathways associated with each canonical axis are coincident, which indicates that the metabolic pathways that were involved in old and recent selection events are similar, although probably with variable intensities and directions [60]. The enrichment analysis identified pathways that are related with immune response (lymphocyte TarBase), muscle development (muscle cell TarBase), protein biosynthesis (translation factors, cytoplasmic ribosomal proteins), skin and pigmentation (epithelium TarBase), glucose metabolism (insulin signaling, integrated pancreatic cancer pathway), fat metabolism (adipogenesis), embryogenesis and morphology (focal adhesion), heart (calcium regulation in the cardiac cell) and uterine metabolism (myometrial relaxation and contraction pathways), regulation of the hypothalamic–pituitary–thyroid axis (TSH signalling pathway), hormonal, cellular cycle (MAPK-signaling pathway, G1 to S cell cycle control, eukaryotic transcription initiation), cell signaling (notch signaling pathway) and extracellular receptors (GPCR, class A rhodopsin-like). Among these, 10 pathways (focal adhesion, integrated pancreatic cancer pathway, adipogenesis, myometrial relaxation and contraction pathways, adipogenesis, lymphocyte TarBase, insulin signaling, MAPK signaling pathway, focal adhesion, epithelium TarBase) had been previously identified in a meta-analysis based on a very large number of studies on selection signatures in cattle [38], which confirmed that the metabolic pathways involved in old and recent processes of selection are similar to those detected by using equivalent approaches in other cattle populations.

**Table 2:** Top 10 enriched pathways for the three axes

Pathway	Ngenes^a^	Total^b^
First axis		
Focal adhesion	48	185
Integrated pancreatic cancer pathway	46	181
MAPK signalling pathway	43	163
Lymphocite TarBase	96	533
Epithelium TarBase	69	340
TSH signalling pathway	25	70
Adipogenesis	36	130
Cytoplasmic ribosomal proteins	27	88
Muscle cell TarBase	77	424
GPCRs, class A rhodopsin-like	53	259
Second axis		
Epithelium TarBase	51	340
Lymphocyte TarBase	62	533
Translation factors	15	51
Focal adhesion	27	185
Adipogenesis	21	130
Muscle cell TarBase	44	424
Notch signalling pathway	11	45
Integrated pancreatic cancer pathway	23	181
G1 to S cell cycle control	14	77
Eukaryotic transcription initiation	10	41
Third axis		
Lymphocyte TarBase	304	533
MAPK signalling pathway	124	165
Insulin signalling	123	163
Muscle cell TarBase	240	424
Calcium regulation in the cardiac cell	116	151
Focal adhesion	132	185
Integrated pancreatic cancer pathway	130	181
Myometrial relaxation and contraction pathways	116	162
Adipogenesis	99	130
Epithelium TarBase	191	340

^a^Ngenes: number of genes present in the genomic regions

^b^Total: number genes in the pathway

## Conclusions

In this study, we confirm that the results of various procedures used to identify signatures of selection varied largely among groups of tests depending on the source of information they use to reject the null hypothesis of absence of selection. However, we observed some correlations between the results of each test. Accordingly, these tests could be clustered into three groups that matched with the three canonical axes of a factor analysis. Moreover, each canonical factor (or group of tests) identified different signals of selection, which were assigned to selection events that occurred at different evolutionary times. In fact, older selection events generated signatures of selection that presented communalities between populations, whereas more recent selection events were detected specifically for each population. Nevertheless, the enriched metabolic pathways associated to each group of tests showed an important degree of agreement which suggests that the traits involved in the selection events were similar during the evolutionary history of the populations.

## Additional files

**Additional file 1: Figure S1.** Manhattan plots of the results along the autosomal genome obtained with the Tajima procedure.

**Additional file 2: Figure S2.** Manhattan plots of the results along the autosomal genome obtained with the Fu and Li procedure.

**Additional file 3: Figure S3.** Manhattan plots of the results along the autosomal genome obtained with the Fay and Wu procedure.

**Additional file 4: Figure S4.** Manhattan plots of the results along the autosomal genome obtained with the SelEstim procedure.

**Additional file 5: Figure S5.** Manhattan plots of the results along the autosomal genome obtained with the XP-CLR procedure.

**Additional file 6: Figure S6** Manhattan plots of the results along the autosomal genome obtained with the *H*12 procedure.

**Additional file 7: Figure S7.** Manhattan plots of the results along the autosomal genome obtained with the *iHS* procedure.

**Additional file 8: Figure S8.** Manhattan plots of the results along the autosomal genome obtained with the nSL procedure.

**Additional file 9: Figure S9.** Manhattan plots of the results along the autosomal genome obtained with the *F*_ST_ procedure.

**Additional file 10: Figure S10.** Manhattan plots of the results along the autosomal genome obtained with the XP-EHH procedure.

**Additional file 11: Figure S11.** Manhattan plots of the results along the autosomal genome obtained with the VarLD procedure.
