# Supplementary figures and images for: On the performance of tests for the detection of signatures of selection: a case study with the Spanish autochthonous beef cattle populations
Source: Genet Sel Evol. 2016 Oct 28;48:81. doi: 10.1186/s12711-016-0258-1 (PMC5084421; doi:10.1186/s12711-016-0258-1)

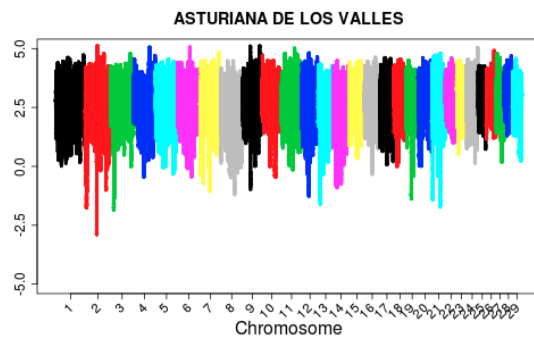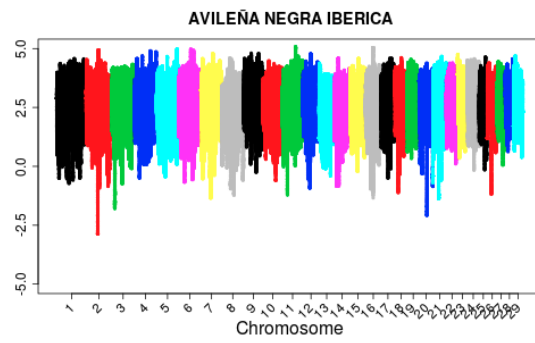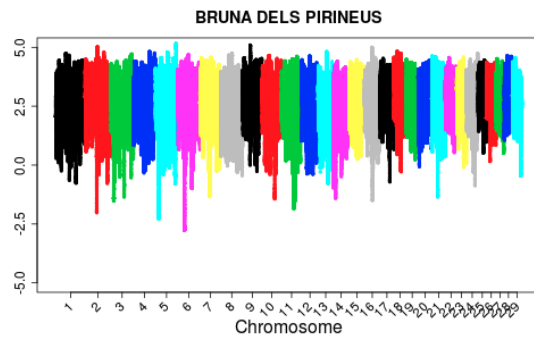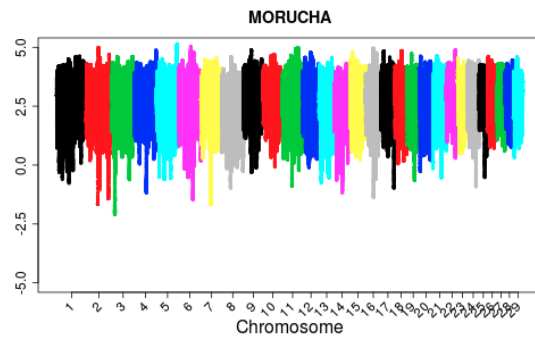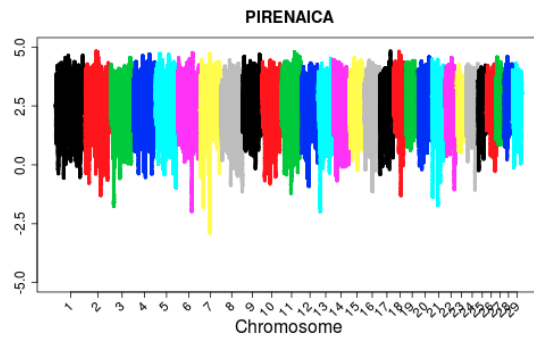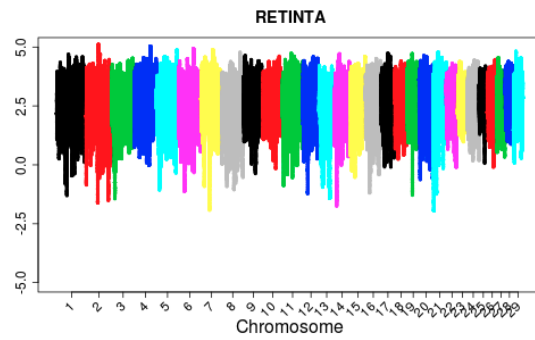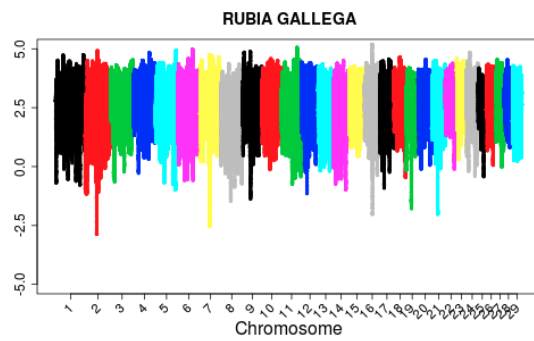

Supplement: Supplementary file 1 — Additional file 1: Figure S1. Manhattan plots of the results along the autosomal genome obtained with the Tajima procedure. [file 12711_2016_258_MOESM1_ESM.pdf]

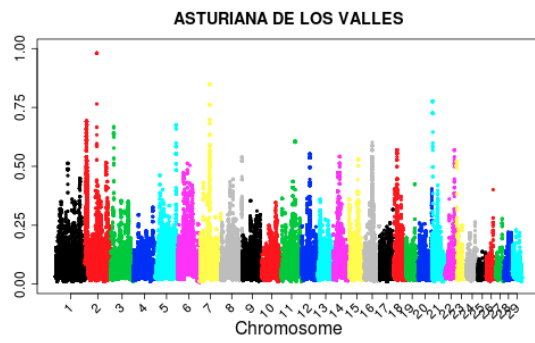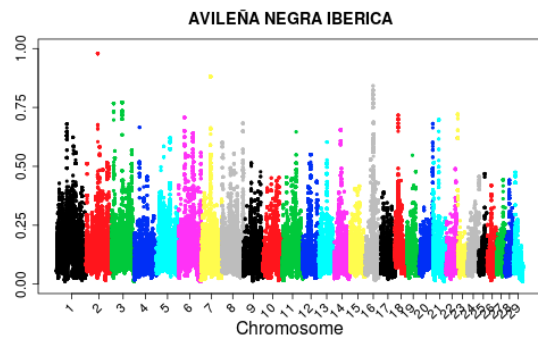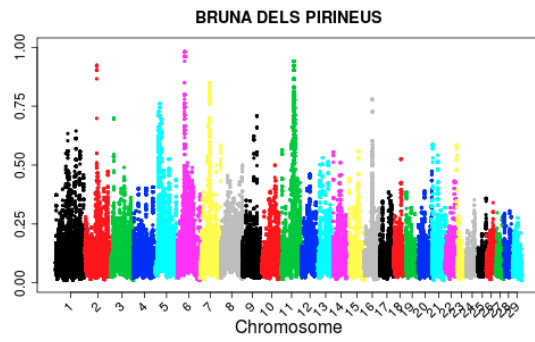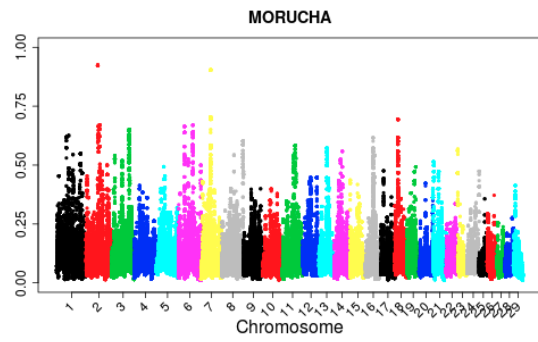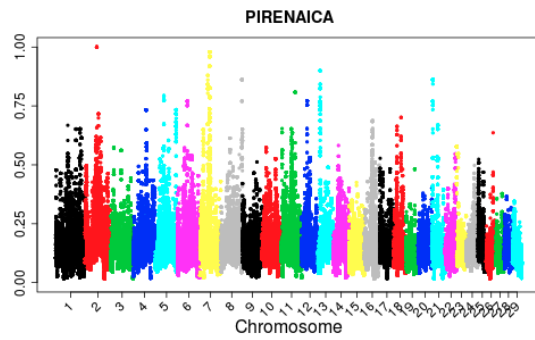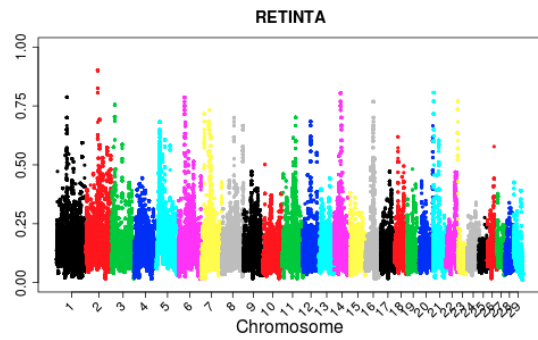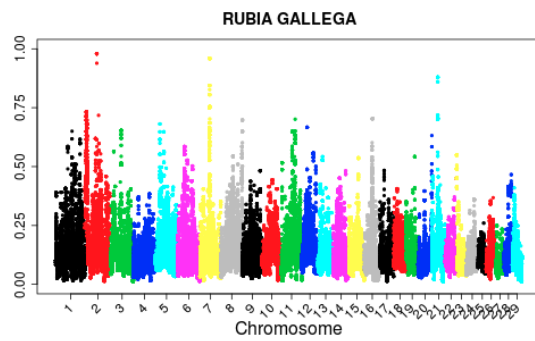

Supplement: Supplementary file 6 — Additional file 6: Figure S6 Manhattan plots of the results along the autosomal genome obtained with the H12 procedure. [file 12711_2016_258_MOESM6_ESM.pdf]

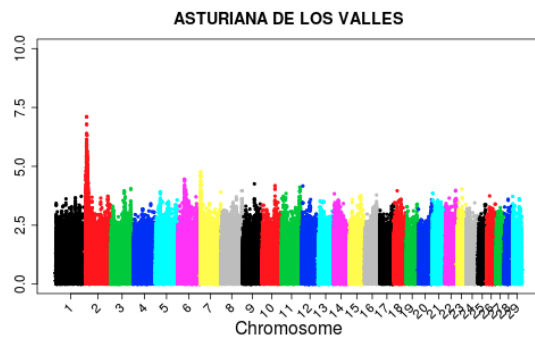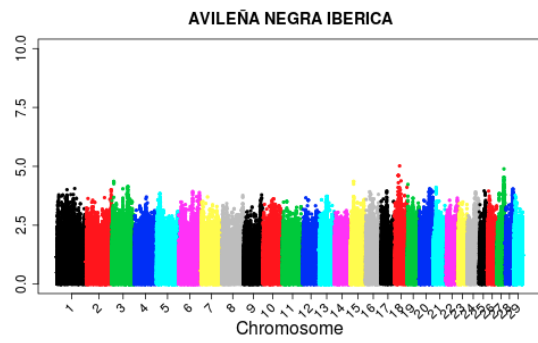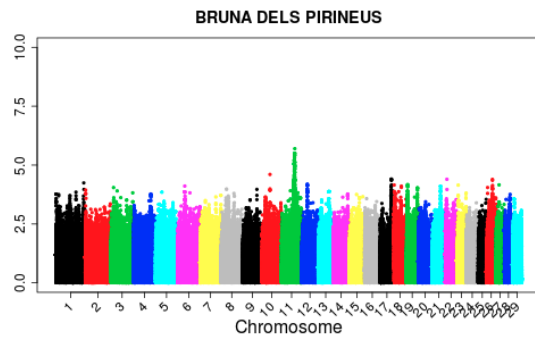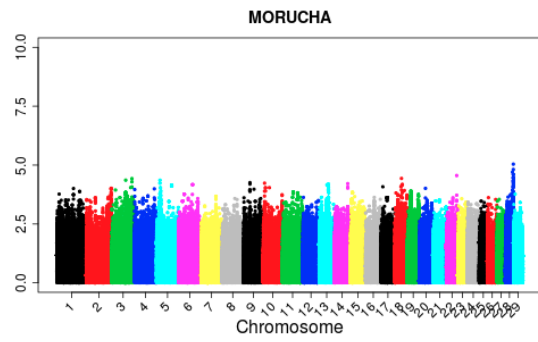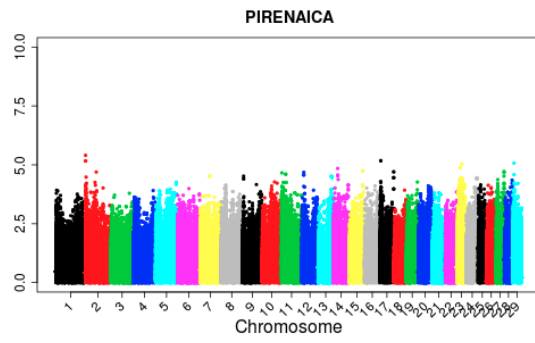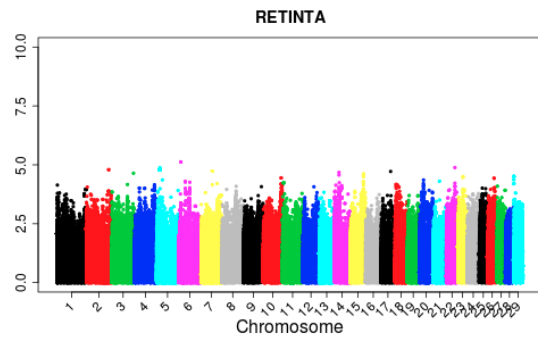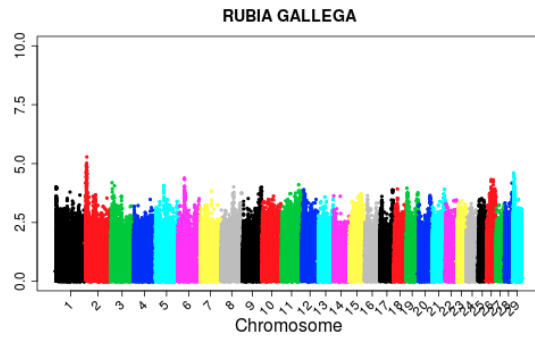

Supplement: Supplementary file 7 — Additional file 7: Figure S7. Manhattan plots of the results along the autosomal genome obtained with the iHS procedure. [file 12711_2016_258_MOESM7_ESM.pdf]

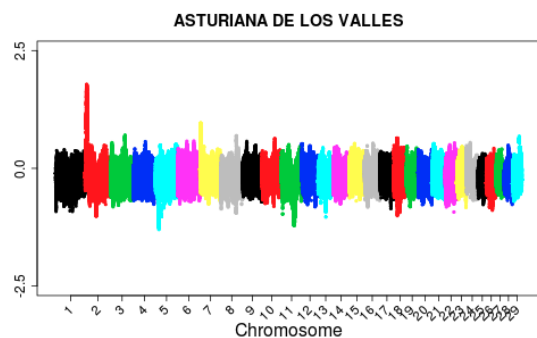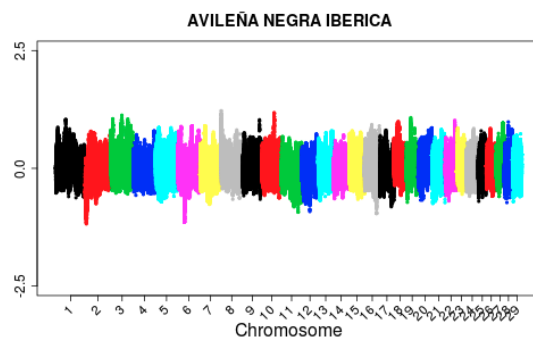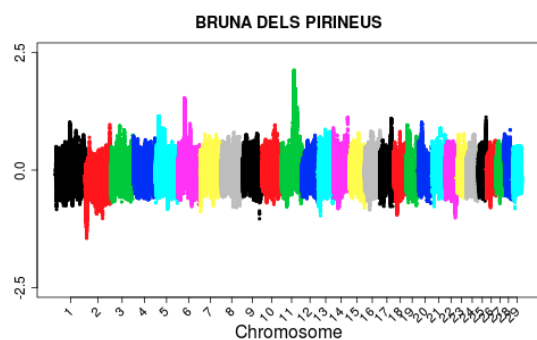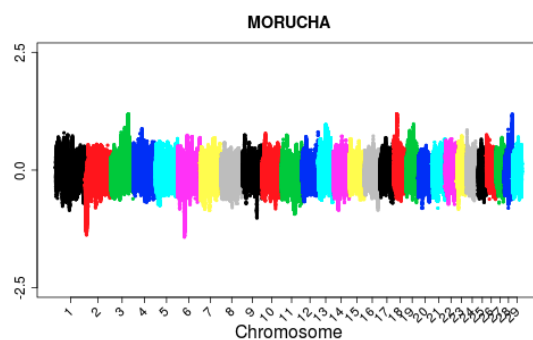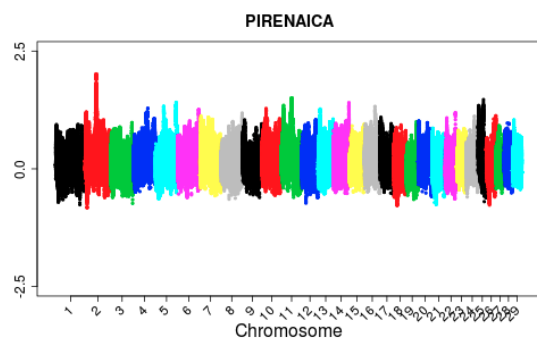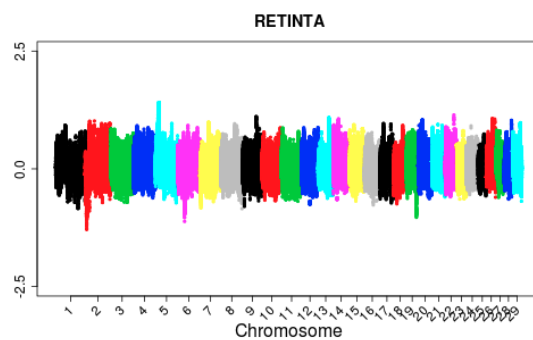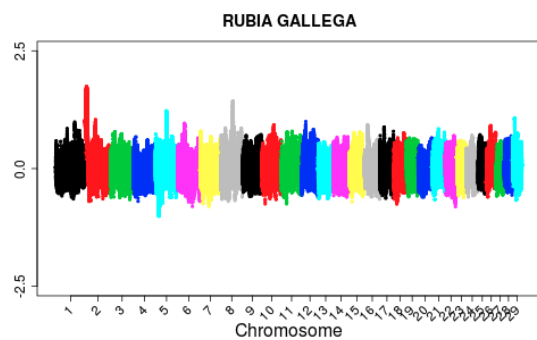

Supplement: Supplementary file 10 — Additional file 10: Figure S10. Manhattan plots of the results along the autosomal genome obtained with the XP-EHH procedure. [file 12711_2016_258_MOESM10_ESM.pdf]
