# Supplementary material for: On the performance of tests for the detection of signatures of selection: a case study with the Spanish autochthonous beef cattle populations
Source: Genet Sel Evol. 2016 Oct 28;48:81. doi: 10.1186/s12711-016-0258-1 (PMC5084421; doi:10.1186/s12711-016-0258-1)
Supplement: Supplementary file 2 — Additional file 2: Figure S2. Manhattan plots of the results along the autosomal genome obtained with the Fu and Li procedure. [file 12711_2016_258_MOESM2_ESM.pdf]

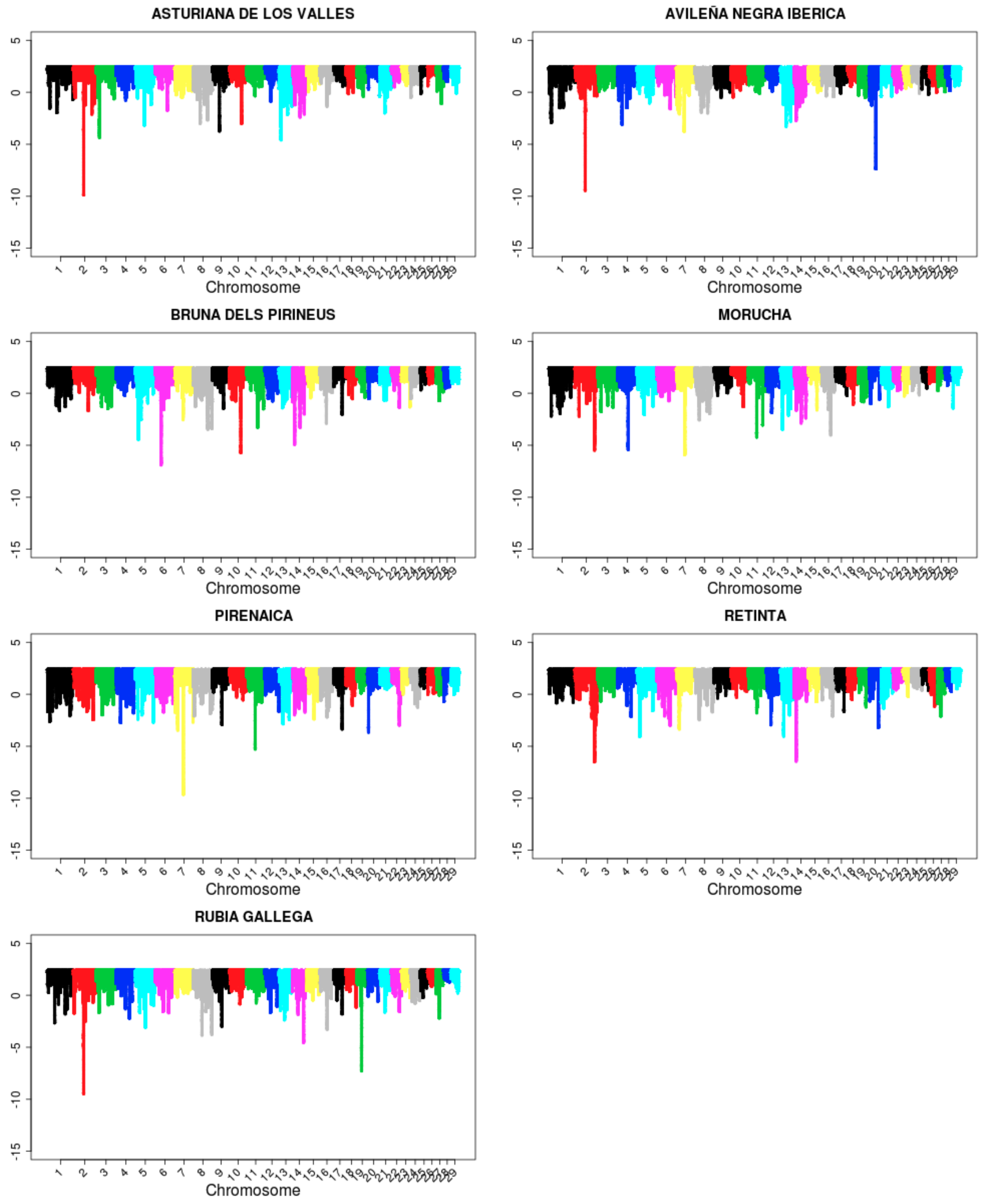
ASTURIANA DE LOS VALLES
AVILEÑA NEGRA IBERICA
BRUNA DELS PIRINEUS
MORUCHA
PIRENAICA
RETINTA
RUBIA GALLEGA
5
0
-5
-10
-15
1 2 3 4 5 6 7 8 9 10 11 12 13 14 15 16 17 18 19 20 21 22 23 24 25 26 27 28 29
Chromosome
