# Supplementary material for: On the performance of tests for the detection of signatures of selection: a case study with the Spanish autochthonous beef cattle populations
Source: Genet Sel Evol. 2016 Oct 28;48:81. doi: 10.1186/s12711-016-0258-1 (PMC5084421; doi:10.1186/s12711-016-0258-1)
Supplement: Supplementary file 4 — Additional file 4: Figure S4. Manhattan plots of the results along the autosomal genome obtained with the SelEstim procedure. [file 12711_2016_258_MOESM4_ESM.pdf]

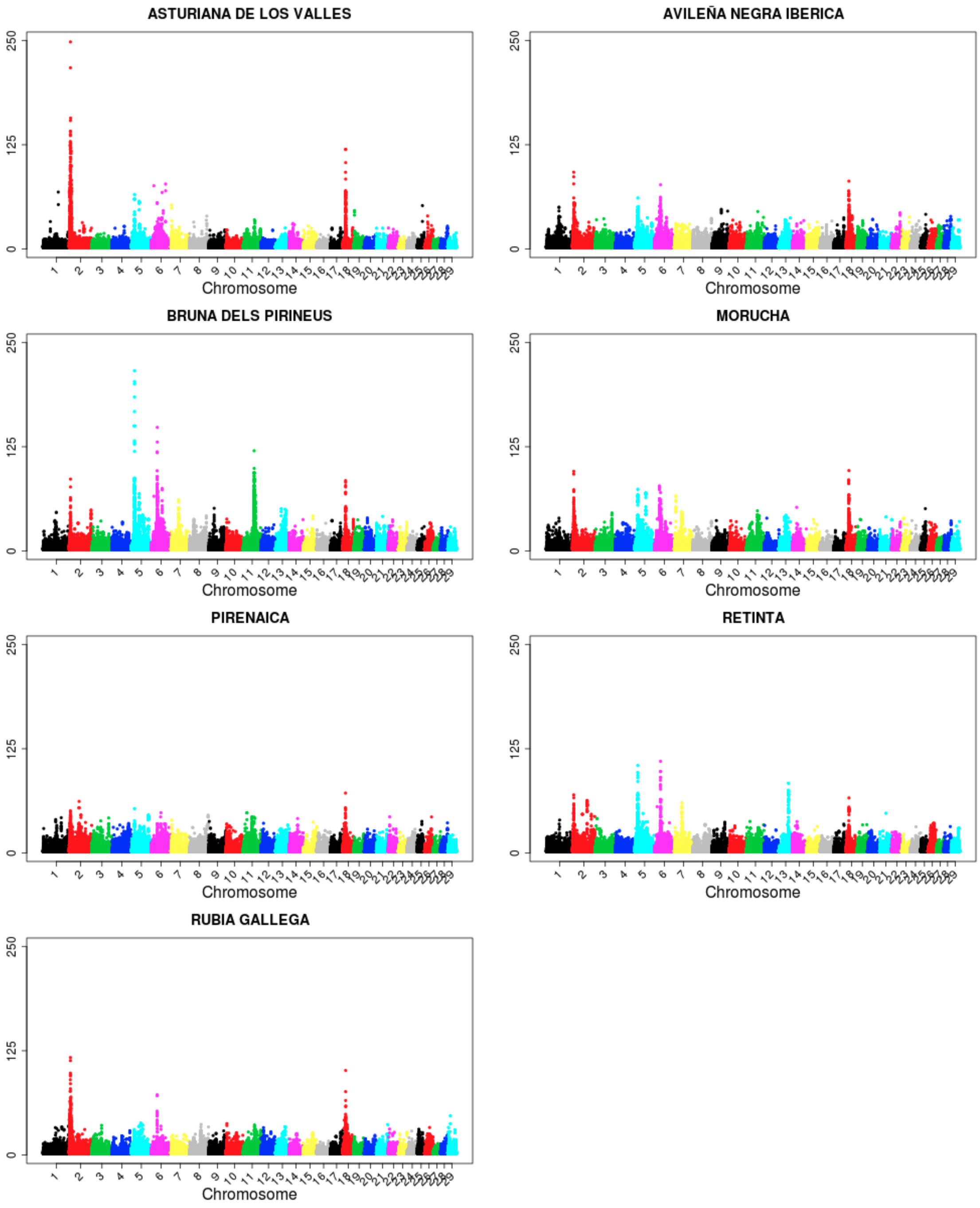
ASTURIANA DE LOS VALLES
AVILEÑA NEGRA IBERICA
BRUNA DELS PIRINEUS
MORUCHA
PIRENAICA
RETINTA
RUBIA GALLEGA
0
125
250
Chromosome
