# Supplementary material for: On the performance of tests for the detection of signatures of selection: a case study with the Spanish autochthonous beef cattle populations
Source: Genet Sel Evol. 2016 Oct 28;48:81. doi: 10.1186/s12711-016-0258-1 (PMC5084421; doi:10.1186/s12711-016-0258-1)
Supplement: Supplementary file 9 — Additional file 9: Figure S9. Manhattan plots of the results along the autosomal genome obtained with the F ST procedure. [file 12711_2016_258_MOESM9_ESM.pdf]

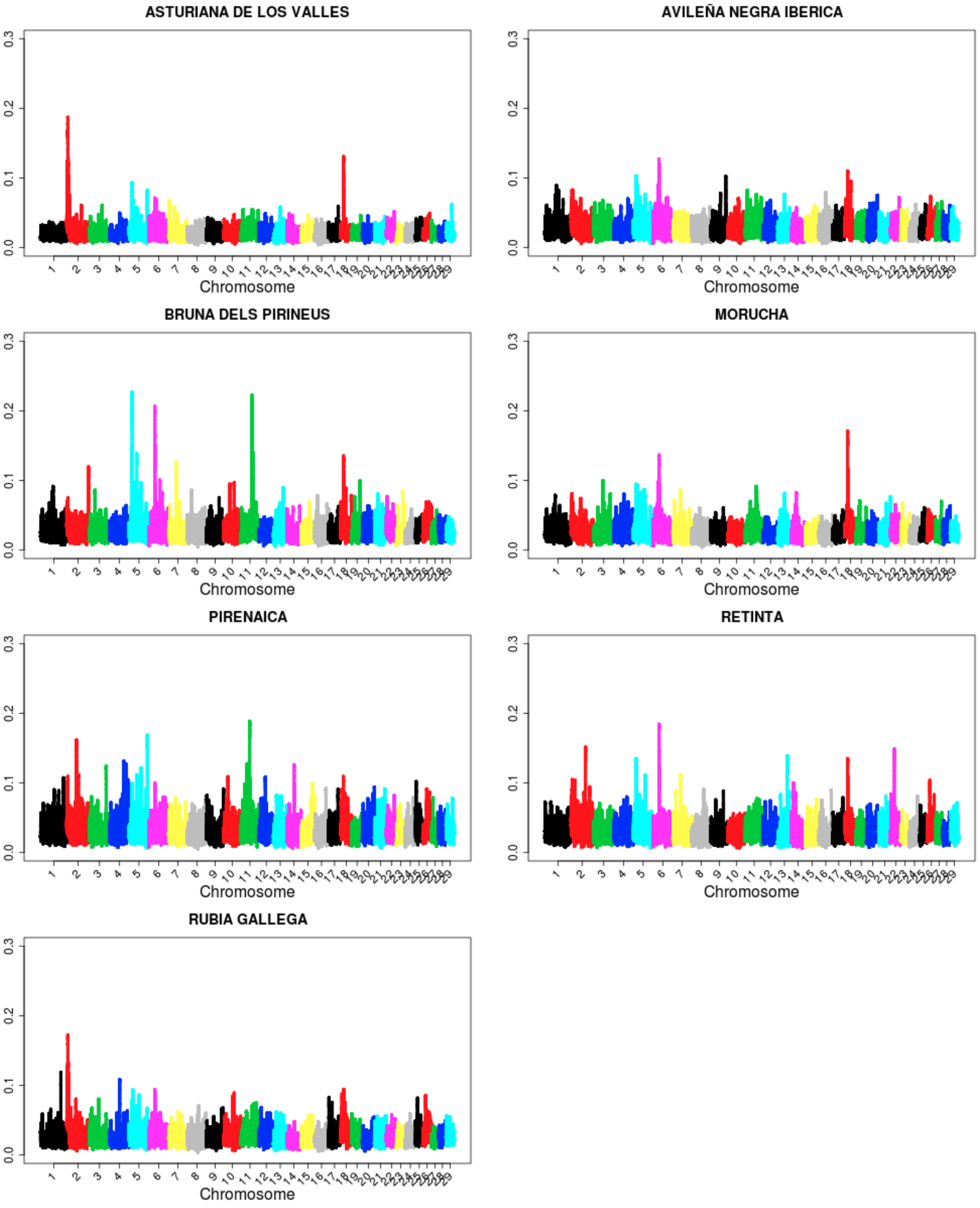
ASTURIANA DE LOS VALLES
AVILEÑA NEGRA IBERICA
BRUNA DELS PIRINEUS
MORUCHA
PIRENAICA
RETINTA
RUBIA GALLEGA
0.0
0.1
0.2
0.3
Chromosome
1 2 3 4 5 6 7 8 9 10 11 12 13 14 15 16 17 18 19 20 21 22 23 24 25 26 27 28 29
