# Supplementary material for: On the performance of tests for the detection of signatures of selection: a case study with the Spanish autochthonous beef cattle populations
Source: Genet Sel Evol. 2016 Oct 28;48:81. doi: 10.1186/s12711-016-0258-1 (PMC5084421; doi:10.1186/s12711-016-0258-1)
Supplement: Supplementary file 11 — Additional file 11: Figure S11. Manhattan plots of the results along the autosomal genome obtained with the VarLD procedure. [file 12711_2016_258_MOESM11_ESM.pdf]

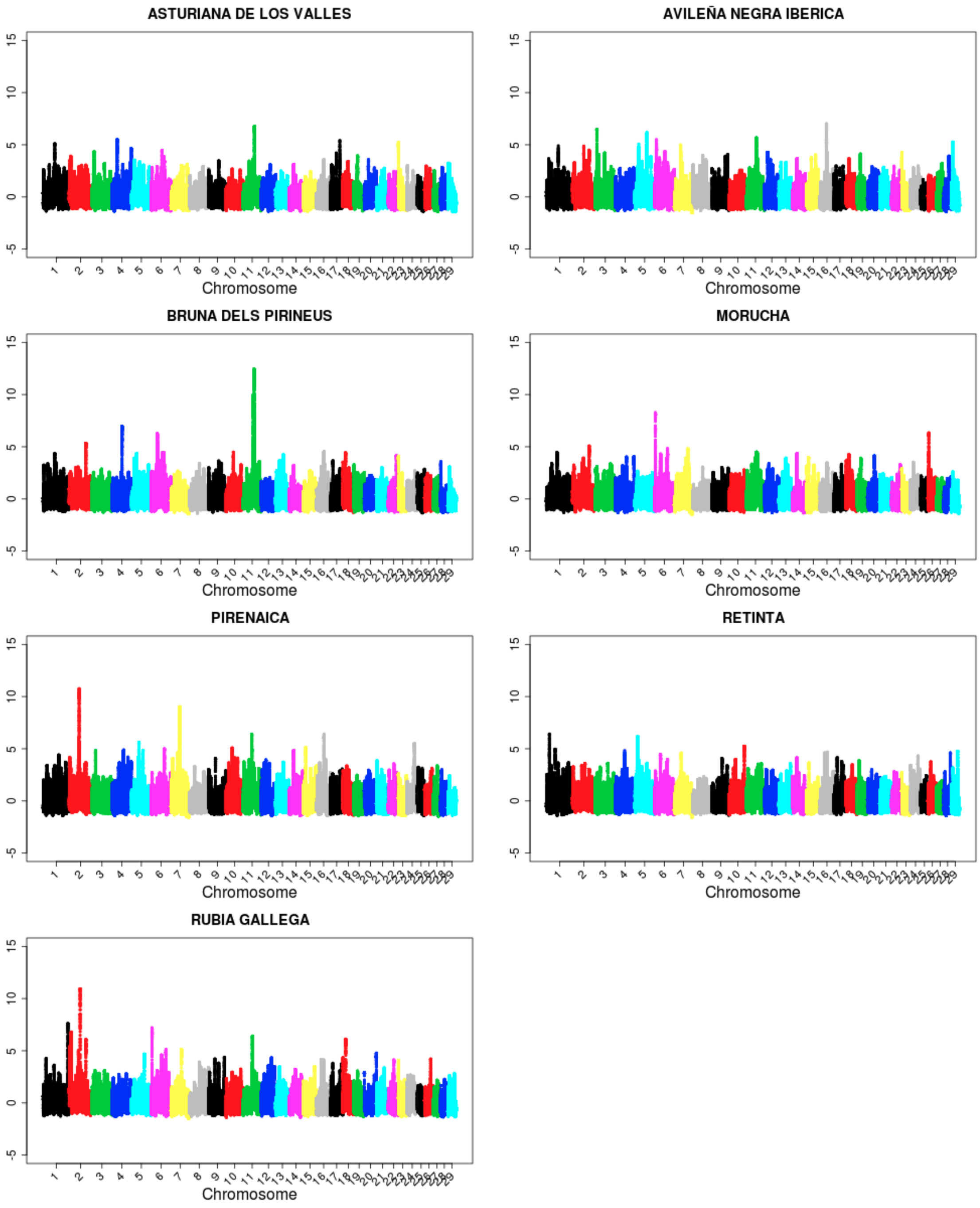
ASTURIANA DE LOS VALLES
Chromosome
AVILEÑA NEGRA IBERICA
Chromosome
BRUNA DELS PIRINEUS
Chromosome
MORUCHA
Chromosome
PIRENAICA
Chromosome
RETINTA
Chromosome
RUBIA GALLEGA
Chromosome
